# Western Dental Society

**Published:** 1858-08

**Authors:** 


[From the Quincy Weekly Whig & Republican.

### MEETING OF THE WESTERN DENTAL SOCIETY.

The above named Society met in this city on Tuesday morning at 11 o'clock, in Lomelino's Hall.

The President, Dr. Isaiah Forbes, of St. Louis, in the Chair. The Society was called to order and a portion of the session was occupied in reading and correcting minutes of previous meeting, and examination of candidates for membership. After which the Society proceeded to the election of officers for the ensuing year.

Dr. W. W. Allport, of Chicago, Ill., was elected President; Dr. I. B. Branch, of Galena, Ill., 1st Vice President; Dr. W. H. Anderson, Hannibal, Mo., 2d Vice President; Dr. I. Forbes,

St. Louis, Mo., Rec. Sec'y; Dr. H. T. Peebles, St. Louis, Mo., Corresponding Sec'y; Dr. A. Blake, Treasurer.

Examining Committee, Drs. Spaulding, McKellops, Forbes, Priest, and Hubbard.

Meeting adjourned until 2 P. M.

At 2 P. M., called to order by President Forbes.

The President elect, after being conducted to the Chair, said:

"GENTLEMEN:—For the honor done me in selecting me as your presiding officer, I return you my sincere and heartfelt thanks.

It is now three years since the "*Western Dental Society*" was first organized, and this is its fourth meeting. Whatsoever of good it may have accomplished toward the advancement of Dental Science, and the making of our profession one of dignity, usefulness and honor, certain it is, that not the least among its useful results, is the better acquaintance and consequent good fellowship of its members with each other.

You will agree with me when I say that this Society has drawn together the better portion of our profession in the West, men who by effort and example would contribute to the advancement of dental knowledge, inciting in them a spirit of emulation and industry which promises much for the future. A spirit of rivalry alike laudable and honorable, doing equal credit to the head and heart.

At the time of the formation of this Society, the expediency of its formation was doubted by many, and its promised usefulness was questioned—but the results that have followed, and which we witness to-day in the numbers, and the interest exhibited by those present, must, I am sure, set at rest the question of expediency in this matter.

The "*Western Dental Society*," has, to day, a *living* existence and I trust will not soon die.

Thanking you again, gentlemen, for the honor you have conferred upon me, I will now discharge the duties of your presiding officer to the best of my ability."

After which and the transaction of some miscellaneous business, the regular subjects of discussion were taken up—the first in order being *Difficult Dentition.*

It was ably discussed by Drs. Spaulding, Allport, McKellops, Blake, Baron, Branch, Sturgiss, Anderson, Hubbard, Anderson, Forbes, and others, developing much of interest to the profession. The discussion was protracted and ably sustained, members speaking more directly to the point than usual on such occasions.

The cause of difficult dentition seemed generally acknowledged as constitutional and generally hereditary. Great reliance was placed upon the use of lime in various forms, as Bone Phosphate, lime water, and free use of the Gum Lancet as remedial agents, with some dissent.

Next in order came a discussion of *Causes and Treatment of Irregularities of the Teeth.*

The discussion was participated in by all the members present, and was interesting and instructive. Cases were reported by Drs. McKellops, Peebles, and Allport, demonstrating clearly that almost any deformity of the face, at all dependent on the teeth or jaws, is under the control of the judicious dentist.

At the close of this discussion, the Society adjourned to meet same place, at 10 A. M., on Wednesday.

SECOND DAY.

Wednesday, 10 A. M.

Convention met pursuant to adjournment, the President in the Chair.

"*Inflamed Dentine*"—and its treatment—was ably, yet briefly discussed—each member stating his particular method and prescriptions; a majority agreeing in the occasional careful use of arsenicum, combined with other substances in various proportions—such as creosote, morphia, tannic acid, alum, &c. One gentleman remarked that recently he treated almost exclusively by applying cold to produce temporary insensibility. All took part and the discussion was lively and interesting.

Next in order came the *Treatment of exposed Nerves.*

Various methods were brought forward as to the practice of the different members—all evincing thought and careful experiment. Cases of capping the nerve were reported, of 20 and 12 years standing, by Dr. Forbes and Dr. Branch; and this practice recommended when the pulp or nerve is in a normal condition. Some, sometimes—and others, always—kill and take out the nerve, fill the cavity and crown. Of this latter class, Dr. Allport reported 184 cases thus treated, of which he has seen 153—of which 8 had small abscesses, and 4 had been extracted—showing conclusively that it is not always best to extract an aching tooth.

SECOND DAY—AFTERNOON SESSION.

First subject for discussion—*Best manner of inserting partial sets with clasps.*

The general opinion seemed to be that this method should be used as little as consistent with success—all admitting its deleterious and destructive tendency on the clasped teeth. That when used, the bearings of clasps should be as few and small upon the teeth as is consistent with proper security to the piece. Various methods were proposed to effect this object, each possessing merit and evincing thought and effort in the right, direction. After which, the relative merits and demerits of different modes of inserting artificial dentures were discussed.

The prevailing preferences seemed to be for Single Gum Teeth, on Gold Plate, and Continuous Gum Teeth. Other methods were favorably spoken of, including Block Work, Vulcanized Rubber and Cheoplasty--some making *fits* with the latter; others *giving it fits.*

A committee on Local Anæsthesia, as used by Dr. I. B. Branch, appointed at the last annual meeting, reported, through their chairman, Dr. Allport, as follows:

The Committee to whom was referred the subject of Local Anæsthesia, by Dr. I. B. Branch's instruments, beg leave to report, that Local Anæsthesia, as produced by congelation, although attended by some inconvenience in its preparation and application, can be employed with gratifying results in a large number of cases. They regard it as particularly useful in cases where the administration of chloroform would not be admissible. W. W. ALLPORT, *Chairman.*

Fifteen minutes were now set apart to present specimens of improvements in dental art, during which many things useful, practical and ornamental were exhibited. It should have been said previously that Dr. Peebles, of St. Louis, read appropriate and instructive papers on the various topics discussed, which were filed with the proceedings.

Dr. McKellops offered the following preamble and resolution:

WHEREAS, owing to the great inconvenience of the officers and soldiers in procuring competent dentists, when necessarily required, and knowing the difficulty in which they are placed, being stationed at distant posts, where it is absolutely impracticable for a regular practitioner of dentistry to visit them—therefore,

*Resolved*, That this Society appoint a committee of five, for the purpose of memorializing Congress on the necessity of appointing dentists to be attached to the regular army, and that we recommend the same to the consideration of the American Dental Convention, and ask their co-operation with us.

The resolution was adopted and the following committee appointed:

Drs. McKellops, Spaulding, Forbes, Branch, and Lewis.

President Allport added by vote.

After some further business incidental to the workings of organized bodies, the Society adjourned to meet at Milwaukee,

Wisconsin, on the first Tuesday in July, 1859. The session, throughout, was characterized by harmony and good feeling. The President, Dr. W. W. Allport, of Chicago, discharged his duties with intelligent promptitude and gentlemanly urbanity, thus doing honor to himself and the Society.

Among the strangers present was Dr. Andrews, of Mississippi, who, by his gentlemanly intelligence in speaking on the various questions, did much to add interest to the meeting. From what we have seen and heard, at this meeting, we are convinced that the Dental Profession includes a large number of men ardently devoted to the advancement of science, in the alleviation of human suffering, who will compare favorably with any other profession extant. And from the amount of theory given, and facts illustrated, we wonder that any in the profession, who desire to excel, can consent to absent themselves from such meetings.

